# HIV-Induced T-Cell Activation/Exhaustion in Rectal Mucosa Is Controlled Only Partially by Antiretroviral Treatment

**DOI:** 10.1371/journal.pone.0030307

**Published:** 2012-01-19

**Authors:** Cesar Mauricio Rueda, Paula Andrea Velilla, Claire A. Chougnet, Carlos Julio Montoya, Maria Teresa Rugeles

**Affiliations:** 1 Grupo Inmunovirologia, Universidad de Antioquia, Medellín, Antioquia, Colombia; 2 Division of Molecular Immunology, Cincinnati Children's Hospital Research Foundation, Department of Pediatrics, University of Cincinnati College of Medicine, Cincinnati, Ohio, United States of America; New York University, United States of America

## Abstract

Peripheral blood T-cells from untreated HIV-1-infected patients exhibit reduced immune responses, usually associated with a hyperactivated/exhausted phenotype compared to HAART treated patients. However, it is not clear whether HAART ameliorates this altered phenotype of T-cells in the gastrointestinal-associated lymphoid tissue (GALT), the main site for viral replication. Here, we compared T-cells from peripheral blood and GALT of two groups of chronically HIV-1-infected patients: untreated patients with active viral replication, and patients on suppressive HAART. We characterized the T-cell phenotype by measuring PD-1, CTLA-4, HLA-DR, CD25, Foxp3 and granzyme A expression by flow cytometry; mRNA expression of T-bet, GATA-3, ROR-γt and Foxp3, and was also evaluated in peripheral blood mononuclear cells and rectal lymphoid cells. In HIV-1+ patients, the frequency of PD-1^+^ and CTLA-4^+^ T-cells (both CD4+ and CD8+ T cells) was higher in the GALT than in the blood. The expression of PD-1 by T-cells from GALT was higher in HIV-1-infected subjects with active viral replication compared to controls. Moreover, the expression per cell of PD-1 and CTLA-4 in CD4^+^ T-cells from blood and GALT was positively correlated with viral load. HAART treatment decreased the expression of CTLA-4 in CD8^+^ T cells from blood and GALT to levels similar as those observed in controls. Frequency of Granzyme A^+^ CD8^+^ T-cells in both tissues was low in the untreated group, compared to controls and HAART-treated patients. Finally, a switch towards Treg polarization was found in untreated patients, in both tissues. Together, these findings suggest that chronic HIV-1 infection results in an activated/exhausted T-cell phenotype, despite T-cell polarization towards a regulatory profile; these alterations are more pronounced in the GALT compared to peripheral blood, and are only partiality modulated by HAART.

## Introduction

During the acute phase of human immunodeficiency virus type 1 (HIV-1) infection, the gastrointestinal-associated lymphoid tissue (GALT) suffers the most substantial immunological and structural damage due to massive elimination of CD4^+^CCR5^+^ T-cells, as a result of high levels of viral replication [1], [2]. This event leads to microbial product translocation from the lumen of the gastrointestinal tract to systemic circulation [3], [4], contributing to the establishment of chronic immune activation [5]. Concomitantly, there is a progressive loss of the regenerative capacity of the lymphoid tissue [6]. Alteration of antigen-presenting cells and T-cells are distinctive; in particular, reduced proliferation and cytokine production by T-cells occurs in response to different stimuli. Many of these defects persist in patients receiving highly active antiretroviral therapy (HAART) [7], [8].

HLA-DR, CD25 and granzymes are molecules associated with activation and effector functions of CD8 T-cells. Indeed, activation of cytotoxic T-cells has been correlated with the control of viral replication, and is one of the best predictors of disease progression [9]. Other markers such as programmed death 1 (PD-1) and the cytotoxic T-lymphocyte antigen 4 (CTLA-4) are classically associated with activation, and persistence of high levels of expression of these markers by peripheral blood T cells of untreated patients is linked to T-cell exhaustion [10]. PD-1 and CTLA-4 upregulation appear closely linked to HIV replication and progressive disease; in fact, specific blockage of these pathways with monoclonal antibodies enhances HIV-1-specific T-cell responses [11], [10].

Although HAART has significantly improved the quality of life of HIV-1-infected patients and particularly their life expectancy, incomplete suppression of viral replication and partial restoration of CD4^+^ T-cells are often seen in GALT, in contrast to peripheral blood, despite continuous use of HAART [12]. Since GALT is a highly regulated tissue and the main site of HIV-1 replication, a detailed phenotypic characterization of its T-cell subsets and their modulation by HAART, is important to better understand HIV-1 pathogenesis.

Considering that GALT disruption induces T-cell activation/exhaustion, in parallel with regulatory processes that are associated with the inability of the immune system to mount effective responses against HIV-1 and other pathogens [13], [14], we were interested in characterizing the immune response in GALT. Our results suggest that HIV-1 infection induces a pattern of T cell activation/exhaustion, affecting both CD4^+^ and CD8^+^ T cells, despite increased polarization towards a regulatory profile. These changes are clearer in GALT than in peripheral blood. Importantly, HAART does not totally normaliza this phenotype.

## Results

### Patient characteristics

As shown in Table 1, groups were matched by age. The macroscopic evaluation of rectosigmoidoscopies was normal in all individuals. No evidence of active opportunistic infections and tumors was detected in any of the HIV-1-infected subjects at the time of sampling. Patients on HAART (H) had been on antiretroviral therapy for a median of 9 (6-13) years; all patients except 2 had an absolute CD4^+^ T-cell count above 300 cells/mm^3^. In this group, 62% (8/13 patients) presented an undetectable viral load (VL; <40 copies/ml), 31% (4/13 patients) had <290 copies/ml, and 1 patient had a VL>1,000 copies/ml (1,193 copies/ml) at the time of sampling. 80% of the untreated patients (U) had CD4 counts >250 cells/mm^3^ and 70% of them had a VL>20,000 copies/ml. As expected, the median VL of the untreated group was significantly higher than that of the treated group (*p*<0.001). The percentage and absolute counts of CD8^+^ T-cells were high in all infected individuals compared to controls (C), with the highest values found in the untreated group (Table 1).

**Table 1:** Characteristics of healthy controls, HAART treated and untreated patients.

	Healthy Controls (n = 10)	HAART Treated (n = 13)	Untreated (n = 10)
Age (years) 1	45 (12)	44 (9)	35 (13)
% Female/Male	50/50	15/85	0/100
CD4^+^ T cell counts (cell/mm^3^) 2	905 (691–1093)	464 (301–1055)	337**(245–677)
CD8^+^ T cell counts (cell/mm^3^) 2	457 (310–618)	1127** (804–1605)	1451***(696–2038)
Viral load (copies/ml)^2^	N/A	40 (40–223)	49430 (19119–88631)

1 :results are expressed as mean (SE);

2 : results are expressed as median (25th–75th percentiles). Groups were compared by the Kruskal-Wallis test and the Dunńs multiple comparison post-test. Significant differences are indicated by

**p<0.01,

***p<0.001. HIV-1 positive patients were compared by U Mann Whitney test p<0.001. N/A: not applicable.

To evaluate the efficacy of HAART on the reconstitution of CD4^+^ T cells, we determined the CD4^+^/CD8^+^ T cell ratio in both peripheral blood and GALT. In controls, the CD4^+^/CD8^+^ T cell ratio in blood was 2.22 (1.22-2.87); however, in normal GALT the frequency of CD4^+^ and CD8^+^ T cells was similar with a ratio of 1.26 (0.63-2.37). On HIV-1+ patients, the depletion of CD4^+^ T cells is clear in both compartments; with a incomplete reconstitution even after prolonged suppressive therapy on PMBC (H:0.47 vs C: 2.22; *p*<0.01) and GALT (H:0.34 vs C: 1.26; *p*<0.01). The CD4^+^/CD8^+^ T cell ratio was strikingly low on the untreated group, with a ratio of 0.30 (0.004-0.69) (U vs C; *p*<0.001) and 0.14 (0.03-0.29) (U vs C; *p*<0.001) in PBMC and GALT, respectively.

### GALT T-cells from untreated HIV-1-infected patients express high levels of molecules associated with immune activation and exhaustion

An important hallmark of chronic HIV-1 infection is the immune activation, which leads to T-cell exhaustion, characterized by the persistent expression by lymphocytes of markers, such as HLA-DR, CD25, PD-1 and CTLA-4. We determined the expression of these molecules in both subpopulations of GALT T-cells and compared it to that of peripheral blood T-cells.

**Figure 1:**
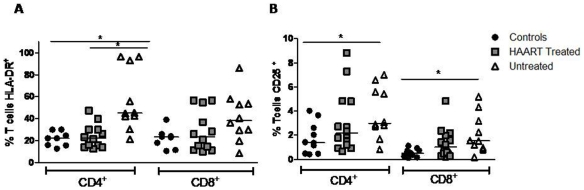
HIV-1 infected patients exhibit higher frequency of activation markers on rectal tissues. Phenotypic characterization of GALT cells from healthy controls, HIV+ HAART-treated and untreated patients. A) Dot blot figures show the frequency of HLA-DR^+^, and B) CD25^+^ on CD4^+^ (left panel) and CD8^+^ (right panel) T-cells. Each bar represents the median of the data. Groups were compared by the Kruskal Wallis test and Dunn's multiple comparisons post-test. Significant differences are indicated by *p<0.05, **p<0.01, ***p<0001.

As shown in Figure 1A left, the frequency of CD4^+^HLA-DR^+^ T cells in GALT was higher in untreated HIV-1-infected individuals than in controls or HAART-treated patients (*p*<0.05). The frequency of peripheral blood CD4^+^HLA-DR^+^ T-cells was higher in both groups of HIV-1-infected individuals compared to controls (median percentage: U: 14.9% vs C: 7.0%; *p*<0.05 and H: 17.2% vs C: 7.0%; *p*<0.00; data not shown). The frequency of CD8^+^HLA-DR^+^ T-cells in GALT was similar in the three groups of individuals (Fig. 1A, right); in peripheral blood, it was higher in untreated patients than in controls (U: 6.2% vs C: 2.4%; *p*<0.05), but similar in controls and HAART-treated patients (data not shown). The frequency of T-cells expressing CD25 showed similar trends, and GALT T-cells from untreated patients exhibited the highest expression (Fig. 1B, *p*<0.05). The frequency of CD25^+^ T-cells in PBMC was similar in all the groups (range 0.7-1.5%; p = 0.82, data not shown).

**Figure 2:**
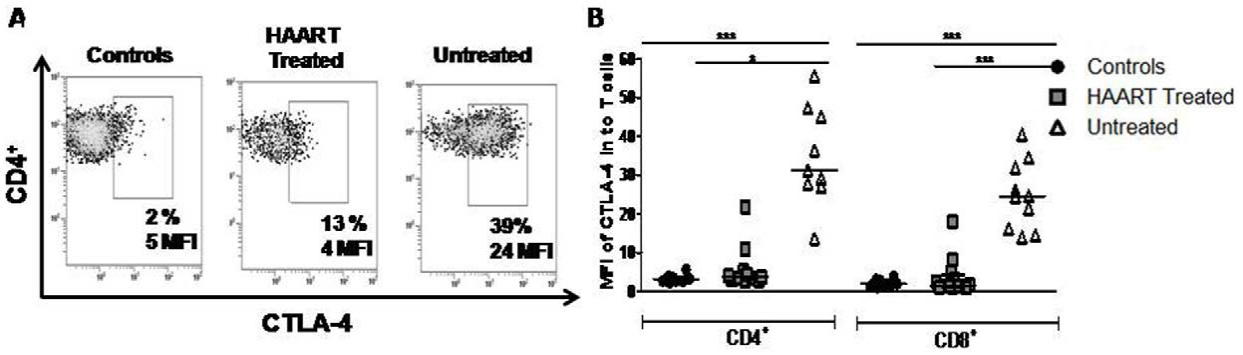
T-cells from untreated infected patients exhibit elevated expression of CTLA-4 in rectal tissues. A) Flow cytometry data from one representative experiment with GALT samples from healthy controls, HIV+ treated and untreated patients are shown. Percentage of CD4^+^CTLA-4^+^ cells and MFI is indicated in each panel. B) MFI of CTLA-4 on CD4^+^ (left panel) and CD8^+^ (right panel) T-cells from GALT.

The frequency of GALT CD4^+^ T-cells expressing CTLA-4 was higher in both HIV-1-infected groups compared to controls, and again the untreated group exhibited the highest values (U vs C; *p*<0.05 and H vs C; *p*<0.001; see Table 2 and representative flow cytometry data shown in Fig. 2A). Untreated patients also exhibited the highest proportion of GALT CD8^+^ T-cells expressing CTLA-4 (U vs C; *p*<0.001 and U vs H; *p*<0.01; Table 2). Expression of CTLA-4 by peripheral blood CD4^+^ and CD8^+^ T-cells showed a trend similar to that observed in GALT samples (data not shown).

**Table 2:** Frequency of CTLA-4^+^ and PD-1^+^ in T cells from GALT of healthy controls, HAART treated and untreated patients.

Frequency of T-cells	Healthy Controls	HAART Treated	Untreated
%CD4^+^CTLA^+^	4.74 (3.43–5.86)	12.74* (5.53–20.61)	28.07*** (19.76–44.16)
%CD8^+^CTLA^+^	1.07 (0.36–1.92)	1.51 (1.26–2.28)	5.33*** ++ (2.80–9.73)
%CD4^+^PD-1^+^	7.75 (5.61–11.01)	19.38* (10.11–33.87)	36.13** (15.33–48.83)
%CD8^+^PD-1^+^	11.17 (2.95–14.85)	9.05 (3.18–16.18)	23.97***(10.02–41.86)

The data are shown as median (25th percentile–75th percentile). Groups were compared by the Kruskal-Wallis test and the Dunńs multiple comparison post-test. Significant differences are indicated by

*p<0.05,

**p<0.01,

***p<0.001 compared to controls and

++ p<0.01 compared with HAART treated group.

When CTLA-4 levels were compared using mean fluorescence intensities (MFI), untreated patients exhibited higher levels of CTLA-4 in both subsets of GALT T-cells compared to controls (*p*<0.001) and to the HAART-treated group (*p*<0.01; Fig. 2B). In peripheral blood, CTLA-4, MFI in CD4^+^ and CD8^+^ T-cells showed a trend similar to that of GALT (median MFI: 25, 3 and 2 in U, H and C respectively; both *p*<0.05).

**Figure 3:**
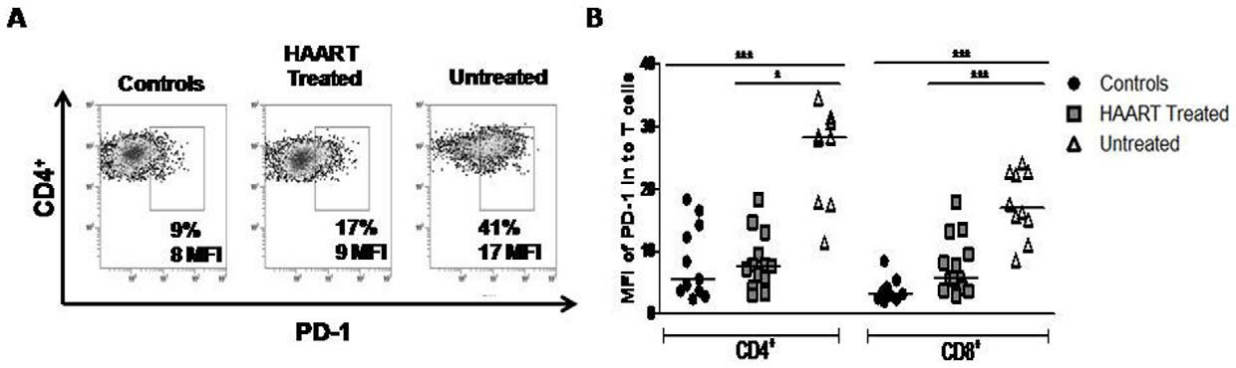
T-cells from untreated patients exhibit elevated expression of PD-1 in rectal tissues. A) Flow cytometry data from one representative experiment with GALT samples from healthy controls, HIV+ HAART-treated and untreated patients are shown. Percentage of CD4^+^PD-1^+^ cells and MFI is indicated in each panel. B) MFI of PD-1 in CD4^+^ (left panel) and CD8^+^ (right panel) T-cells from GALT.

The frequency of GALT CD4^+^ T-cells expressing PD-1 was significantly higher in both groups of HIV-1 infected individuals than in controls, (U vs C; *p*<0.05 and H vs C; *p*<0.01; Table 2; a representative example of staining is shown in Fig. 3A). The frequency of GALT CD8^+^PD-1^+^ T cells was also significantly higher in HAART naïve compared to control cells (U vs C; *p*<0.001; Table 2). In contrast, in peripheral blood, the frequency of CD4^+^ (range 2.6–4.9%) and CD8^+^ (range 2.1–2.5) T-cells expressing PD-1 was similar among the groups (data not shown).

**Figure 4:**
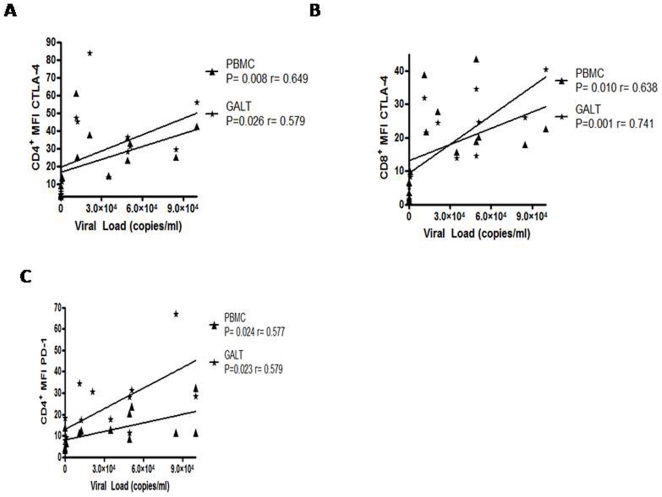
Phenotypic alterations in T-cells are modulated by HIV-1 viral load. Spearman correlation between plasma viral load and phenotype of T cells from PBMC and GALT. Positive correlation between plasma viral load and CTLA-4 expression. A) In CD4^+^ and B) CD8^+^ T cells. C) Positive correlation between viral load and PD-1 expression on CD4^+^ T cells.

Since the levels of PD-1 per cell, rather than the percentage of cells expressing PD-1, has been shown to correlate with T-cell exhaustion [11], [15], the PD-1 MFI was determined in all compartments. PD-1 MFI in both GALT T-cell subsets was higher in patients with higher viral load than in controls (*p*<0.001) or treated patients (*p*<0.01; Fig. 3A and 3B). Similar data were found for PD-1 MFI in peripheral blood T-cells (median MFI: 16, 4.5 and 3.5 for U, H and C, respectively; both *p*<0.01). To evaluate the strength of the association between the T-cell phenotype and viral load, correlations were examined in patients with detectable viral load. Interestingly, the CTLA-4 MFI in CD4^+^ and CD8^+^ T-cells from PBMC and GALT was positively correlated with viral load (Fig. 4A and 4B). Similarly, the PD-1 MFI in CD4^+^ T cells from the blood and GALT was positively correlated with viral load (Fig. 4C). Similarly, a positive correlation between the viral load and %CD8^+^PD-1^+^ (r = 0.53; *p* = 0.0008), %CD4^+^CTLA-4^+^ (r = 0.62; *p* = 0.0016) and %CD8^+^CTLA-4^+^ (r = 0.66; *p* = 0.0005) on GALT was also observed. In contrast, no significant relationships for VL and % PD-1/CTLA-4 T cells in peripheral blood were seen.

### Decreased expression of granzyme A by GALT CD8^+^ T cells from untreated patients

**Figure 5:**
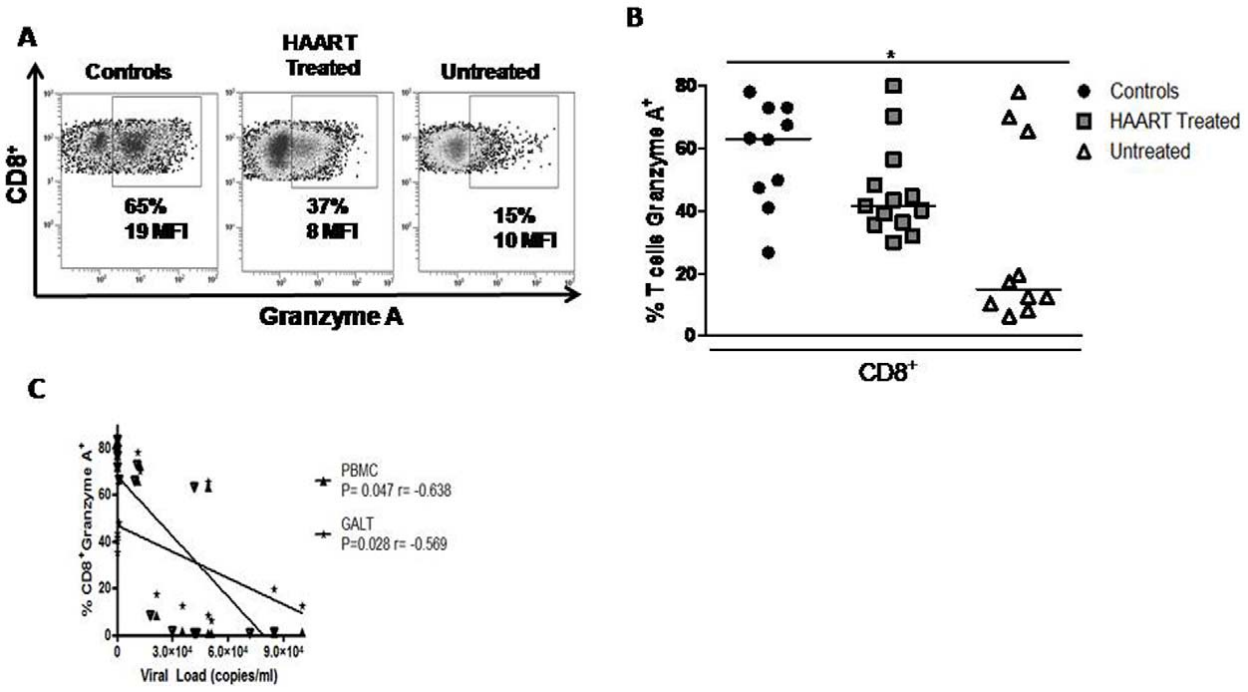
Decreased expression of granzyme A by CD8^+^ T cells in GALT from untreated patients. A) Flow cytometry data from one representative experiment shows the frequency of CD8^+^ granzyme A^+^ from healthy controls, HAART-treated and untreated patients. B) Frequency of total participants of CD8^+^ granzyme A^+^ in GALT. C) Frequency of CD8^+^ granzyme A^+^ T-cells was inversely correlated with viral load.

Granzyme A, a molecule associated with cytotoxic function, has been shown to correlate with CD8 protective function [16]. The frequency of granzyme A^+^ CD8^+^ T-cells was significantly lower in PBMC (data not shown) and GALT from HAART naïve group than in controls (Fig. 5A and 5B). Interestingly, normal frequency of CD8^+^ granzyme A^+^ T-cells was observed in three untreated individuals, who also exhibited the lowest viral load within this group (<20,000 copies/ml). In fact, the frequency of CD8^+^ granzyme A^+^ T cells in blood or GALT was inversely correlated with viral load (Fig. 5C).

### Treg subsets are altered in GALT and blood of untreated patients

**Figure 6:**
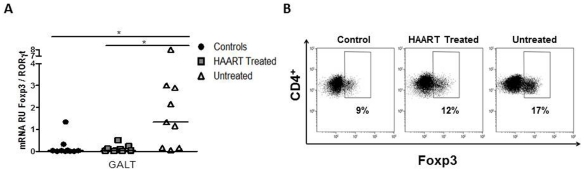
Alteration of the expression of Foxp3 and ROR- γt **ratio in cells from untreated patients.** A) Relative units (RU) of ROR-γt and Foxp3 mRNAs were measured by qRT-PCR. Whisker boxes figures show the ratio of RU of Foxp3/ROR-γt in GALT from healthy controls, HAART-treated and untreated patients. B) Flow cytometry data from one representative experiment show the expression of Foxp3 on CD4^+^ T cells in healthy controls, HAART-treated and untreated patients.

Finally, to evaluate the potential variations of the CD4^+^ T-cell subpopulations in GALT, the mRNA expression of transcription factors specific for Th1 (T-bet), Th2 (GATA-3), regulatory T-cells (Treg; Foxp3), and for Th17 (ROR-γt) cells was measured. mRNA Foxp3 expression in GALT was significantly increased in untreated HIV-1-infected patients compared to HAART-treated subjects and controls (U vs H; *p*<0.05 and U vs C; *p*<0.05; Table 3). Expression of the other transcription factors was similar among the groups (Table 3). Changes in Foxp3 expression led to changes in the Foxp3/ROR-γt mRNA ratio, which was significantly higher in the GALT of untreated patients compared to controls and treated patients (both *p*<0.05; Fig. 6A). HAART normalized this ratio, both in the GALT (Fig. 6A) and PBMC (not shown). In addition, the GALT Foxp3/ROR-γt ratio was positively correlated with viral load (r = 0.472, *p* = 0.035).

**Table 3:** Expression of transcription factors in gastrointestinal tissue of controls, HAART treated and untreated patients.

Transcription factor	Healthy Controls	HAART Treated	Untreated
Foxp3	0.006 (0.002–0.010)	0.005 (0.003–0.010)	0.029*^Φ^ (0.028–2.153)
ROR-γt	0.147 (0.070–0.325)	0.136 (0.0957–0.178)	0.166 (0.079–0.188)
T-bet	0.008 (0.005–0.0152)	0.009 (0.005–0.014)	0.027 (0.002–0.490)
GATA-3	0.129 (0.069–0.445)	0.160 (0.122–0.275)	0.034 (0.013–0.577)

Data are expressed as relative units after normalization on UB2D2 expression. Median (25th-75th percentiles) are shown for each transcription factors. Groups were compared by the Kruskal–Wallis test and the Dunńs multiple comparison post-test. Significant differences are indicated by * ^Φ^ p<0.05 among untreated and controls (*) and HAART treated (**^Φ^**).

To corroborate our data, the expression of Foxp3 protein in CD4^+^ T cells was determined by flow cytometry in biopsies of three individuals per group. As shown in the representative figure 6B, the frequency of Treg (CD4^+^Foxp3^+^) was increased in untreated patients compared to controls (U: 22,21% vs C: 8.26% ; *p*<0.05) (data not shown). To rule out the possibility that the increase of Foxp3 was due to cell activation rather that an increase in Treg frequency, additional markers associated with Treg cell phenotype were evaluated. The CD4^+^Foxp3^+^ cells were also CD25^+^ and CD127^Low/-^ (data not shown). Moreover, the Treg/CD4^+^ ratio in controls, HAART treated and untreated patients was 1.01 (0.66–2.59), 3,89 (3.29–6.27) and 5.26 (3.48–9.32) respectively. This ratio was significantly higher in untreated patients compared with controls (U vs C; p<0.05). Although not significant, in HAART treated patients the Treg frequency (14.32%) and Treg/CD4^+^ ratio were also increased. Unfortunately, it was not possible to detect IL-17 protein by flow cytometry.

## Discussion

The GALT is an organ continually exposed to food and microbial antigens, conditions that promote distinct functional responses and phenotype of gut lymphocytes. During acute HIV-1 infection, viral replication induces severe immune and structural damages in GALT, affecting the gut mucosal barrier, promoting translocation of different microbial components to the systemic circulation, which is considered as one of the main mechanisms underlying chronic immune activation [17]. Since the pathophysiological findings that occur during HIV-1 infection have been mainly characterized in circulating immune cells, we focused our study on GALT T-cells. As previously reported [12], [18], both the proportion and the absolute numbers of CD4^+^ T cells decreased in the peripheral blood and GALT of HIV-1+ patients, including in HAART-treated patients. This finding could be explained by incomplete viral suppression in GALT [19], but it could be also associated with the immune hyper-activation state exhibited by HIV-1 infected individuals [20], [21] .

T-cell chronic activation induced by antigen persistence can result in a functional unresponsive phenotype, known as immune exhaustion [22]. This state is characterized by the loss of effector functions such as proliferation, cytokine secretion and cytotoxic activity [10], [22], [23]. T-cell exhaustion is observed during HIV-1 infection; and previous reports have shown higher levels of CTLA-4 or PD-1 (protein or mRNA) on circulating CD4^+^ and CD8^+^ T-cells from HIV-1 infected patients, compared to uninfected individuals [10], [14], [24]. Here, we show that GALT CD4^+^ and CD8^+^ T cells generally expressed higher expression of molecules associated with exhaustion than peripheral T- cells, but this upregulation was particularly striking in HIV-infected individuals. Indeed, the frequency of PD-1 and CTLA-4-expressing T-cells was about two-fold higher in GALT than PMBC in controls and four-fold higher in HIV-1-infected individuals, even after treatment. Several mechanisms could explain these findings: i) breakdown of the gastrointestinal mucosal barrier lead to translocation of luminal lipopolysaccharide (LPS) and bacterial DNA, triggering a sustained immune activation [17], [25], and ii) persistent exposure to HIV antigens drive this activation, which could be particularly intense in the main sites of HIV replication such as the GALT [22]. The second mechanism may be the most important, as HIV-1- or SIV-specific CD8^+^ T-cells are those expressing the highest levels of PD-1 [26], [27]. Our data also supports such hypothesis as high frequencies of mucosal CD4^+^ and CD8^+^ T-cells expressing CTLA-4 or PD-1 were detected in HIV-1-infected patients with or without control of viral replication compared to those seen in uninfected controls. Expression levels of these molecules were also significantly higher in untreated patients. Moreover, viral load was correlated with the expression of the inhibitory molecules CTLA-4 and PD-1, suggesting a vicious cycle where HIV-1 and immune alterations persist during the chronic phase of infection.

Interestingly, the expression of PD-1 and CTLA-4 was always higher in CD4^+^ than in CD8^+^ T- cells, suggesting the existence of additional mechanisms inducing these molecules in CD4^+^ T- cells. These could include interactions with HIV-1-exposed dendritic cells (DCs) or direct signaling by the HIV-1 Env proteins as both pathways have been shown to induce PD-1 or CTLA-4 expression [14], [28]. Alternatively, the protein Nef induces PD-1 in HIV-1-infected CD4^+^ T-cells [29].

Similarly to a previous report [30], a strikingly low level of granzyme A expression by peripheral and GALT CD8^+^ T-cells from HIV-1-infected patients was observed, particularly in untreated patients. In contrast, other studies have reported an increased expression of granzyme A by HIV-specific CD8^+^ T-cells in lymphoid tissues such as tonsils, lymph nodes and GALT from untreated patients [16], [31], [32]. One possible explanation for this apparent contradiction is the fact that the later studies recruited only individuals expressing the HLA A*0201 allele, which is associated with natural resistance to HIV-1 infection and slow progression [33]. HAART partially normalized defective granzyme expression, since the frequency of CD8^+^ T-cells expressing granzyme A was not significantly different in treated patients and uninfected controls.

To further explore the cytokine profiles during HIV-1 infection, we measured the expression of the transcription factors reported to be associated with different T-cell lineages, i.e. T-bet, GATA-3, Foxp3 and ROR-γt that are expressed by Th1, Th2, Treg and Th17 respectively. In most cases, a GALT Th17 response has been associated with lower pathogenicity [34]. In contrast to another study [35], we did not observe a decrease in Th17 expression in the GALT of HIV-infected patients. This difference may have resulted from the low number of subjects enrolled in our study and/or methodological differences, since the previous report [35] evaluated the expression of IL-17 protein and we quantified ROR-γt mRNA. One limitation of our approach is that it does not allow analyzing the plasticity of the Treg and Th17 subsets, the simultaneous expression of Foxp3 and ROR-γt molecules in some cells [36], [37]. Treg activation in presence of proinflammatory cytokines induce the production of IL-17 [36], [37]; therefore we cannot rule out the possibility that the similar ROR-γt expression in patients and controls is due to the increased frequency of Treg exhibiting an intermediate differentiation profile (CD4^+^CD25^Hi^Foxp3^+^ROR-γt^+^) in the HIV-infected groups. This subpopulation has not yet been reported in HIV-infected patients, and this would deserve further investigation.

A higher Treg frequency was found in lymphoid tissues and peripheral blood of HIV-1-infected patients, which was correlated with disease progression in chronically infected patients [13], [14], [38]. We found a change in the Foxp3/ROR-γt balance in PBMC and GALT from the untreated patients, which was due to increased Foxp3 RNA levels. Increased Foxp3 expression was confirmed by flow cytometry. This result is in agreement with previous reports [13], [39], and supports the hypothesis that HIV-1 infection favors the Treg lineage. Mechanisms underlying such changes are not well established, but could include Treg expansion, migration, increased survival following CD4-gp120 interaction and/or peripheral non-Treg conversion, particularly in the GALT [13], [14]. Of note, PD-1 and CTLA-4 expression inhibit IL-2 and IFN-γ production but increase the enzyme indoleamine 2,3-dioxygenase (IDO) expression by DC [40], [41], [42], conditions that would favor Treg polarization [13], [43], [44], [45]. Further studies are required to determine whether mucosal Treg are functional. Of interest, Shaw et al recently showed accumulation of highly activated Treg in rectal mucosal tissues, and that these cells were suppressive [46].

Increased Treg frequency could thus be considered, along with increased frequency of activated T cells, as a predictive value for disease progression. However, although Treg appear fully functional during HIV infection, as evidenced in suppressive assays performed ex vivo [46], [47], [48], [49], they are not able to control excessive immune activation [39]. These data could be explained by a so-far unrecognized deficit in Treg functionality. Alternatively, it is possible that Treg frequency increases over time to quell this ongoing inflammation [50], but is able to control it, not abolish it. Interestingly, similar dynamic appears at play in aging, in which increased frequency of functional Treg [51] does not completely control the pro-inflammatory environment (the "inflamm-aging") [52], [53]. Future studies will be needed to clarify this important issue.

In conclusion, our results suggest that expression of activation/exhaustion markers by T cells is increased during HIV-1 infection, particularly in the GALT, and is associated with increased polarization towards a regulatory profile. These alterations are not completely restored by HAART. Our findings thus imply that other therapeutic strategies besides HAART will be necessary in order to restore functional immune responses in the GALT of HIV-infected patients.

## Materials and Methods

### Subjects and sample collection

Three groups of subjects, living in the metropolitan area from Medellin-Colombia, were included in the study: healthy controls (C, n = 10), chronic HIV-1-infected patients on HAART treatment (H, n = 13), and untreated (U n = 10) chronic HIV-1-infected patients with viremia greater than 10,000 copies/ml. All the patients have at least one year of HIV confirmed diagnosis.

The demographic and clinical characteristics of these individuals are shown in Table 1. Absolute CD4^+^ T-cell numbers were calculated in whole blood samples. Plasma HIV-1 RNA levels were assessed by RT-PCRq (COBAS AmpliPrep/COBAS TaqMan). All individuals signed an informed consent approved by the Bioethical Board for Human Research of the University of Antioquia, prepared according to the Colombian legislation (Resolution 008430, 1993).

### Isolation of cells from peripheral blood and GALT

Peripheral blood mononuclear cells (PBMC) were obtained from 10 ml of heparin venous blood samples by centrifugation on Histopaque (Sigma-Aldrich, St Louis, MO, USA) for 30 min at 400 x g. Rectal tissue was obtained by rectosigmoidoscopy from the rectum at 10 cm from the anal verge. A flexible sigmoidoscope with a single endoscopy biopsy forcep FB-24K-1 (Olimpus America Corp, Melville, NY, USA) was used. At each procedure, 20 tissue samples of ∼3 mm were obtained. Samples were processed by digestion using collagenase type II from *Clostridium histolyticum* (Sigma) at 0.5 mg/ml diluted in RPMI 1640 and 7.5% FCS (fetal calf serum) (penicillin 100 U/ml, streptomycin 100 µg/ml, amphotericine B 0.25 µg/ml) (Gibco-BRL; Grand Island, NY, USA), during 30 min at 37°C while shaking. After collagenase digestion, biopsy fragments were further disrupted by repeated passage through a 30 ml syringe with a blunt ended 16 gauge needle (Stem Cell Technologies, Vancouver, BC). Rectal cells (RC) were isolated from the fragments by passage through a nylon strainer of 70 µM (Falcon, Lincoln Park, NJ, USA). PBMC and RC were washed with Dulbecós PBS (DPBS) (Sigma-Aldrich; St Louis, MO, USA) to remove excess histopaque and collagenase.

### Antibodies

The following anti-human monoclonal antibodies were used: anti-CD4-ECD (clone SFCI12T4D11) (Beckman Coulter Fullerton, CA, USA); anti-CD3-PE-CY7 (clone UCHT1); anti-HLA-DR-PE (clone LN3); anti-CD25-PE-CY5 (clone BC96); anti-PD-1-PE (CD 279, clone MIH4), anti-Foxp3-FITC (clone PCH101) (e-Bioscience; San Diego, CA, USA); anti-CD3-FITC (clone UCHT1); anti-CD4-PE-Cy5 (RPA-T4); anti-CD8-PE (RPA-T8); anti-CTLA-4 PE (CD 152, clone BNI3) and anti-Granzyme A-PE (clone MOPC-21) (BD Pharmingen; San Diego, CA, USA). Appropriate isotype controls were used for each antibody.

### Flow cytometric analysis of whole blood and isolated rectal cells

For CD4+ T cell count, 100 µl of whole blood samples were incubated for 30 min at 4°C with anti-CD3, anti-CD4 and anti-CD8 monoclonal antibodies; red blood cells were eliminated by lysis buffer (BD Biosciences) and washed twice with DPBS before analysis by flow cytometry.

PBMC and RC were treated with 20 µg/ml of human IgG (Sigma) to block Fc receptors, and stained on the surface with anti-CD3, -CD4, -CD25 or -PD-1 for 30 min at 4C. The cells were washed with DPBS, then fixed/permeabilized (Foxp3 staining buffer kit, e-Bioscience) and stained intracellularly using monoclonal antibodies against CTLA-4, Foxp3 or granzyme A for 30 min at 4C. Also, the expression of activation markers such as HLA-DR and CD38 was evaluated on whole blood samples using specific monoclonal antibodies. At least 100,000 events were acquired for each sample on the lymphocyte region, using the flow cytometry FC500 (Beckman Coulter) and analyzed using the Kaluza Software (Beckman Coulter). T-cells were gated first based on forward- and side-scatter properties, then as CD3^+^CD4^+^ and CD3^+^CD8^+^ cells. To define the limit of the positive gate, a CD3^-^ population was used as negative reference; the MFI was analyzed in the positive population.

### Foxp3, ROR-γt, T-bet and GATA-3 mRNA expression

Rectal biopsies were placed in RNAlater (Qiagen, Inc., Valencia, CA, USA) and stored at −70°C for subsequent RNA extraction. RNA was isolated with the quiazol reagent (Invitrogen Corp., Carlsbad, CA, USA); 1 µg of RNA was used for complementary DNA (cDNA) synthesis with RT-PCR using oligo (dT) and random hexamers by the SuperScriptIII single stranded cDNA synthesis kit (Invitrogen). The cDNA obtained was diluted 1:3 and used in quantitative RT-PCR reactions using SYBER Green (qPCR Master Mix kit, Fermentas Life Sciences, Hanover, MD, USA) with the following primers: Foxp3 (Forward: 5-CAGCACATTCCCAGAGTTCCTC-3; Reverse: 5-GCGTGTGAACCAGTGGTAGATC-3)*;* RORγt (Forward: 5-TTTTCCGAGGATGAGATTGC-3; Reverse: 5- CTTTCCACATGCTGGCTACA-3); T-bet (Forward: 5-GCCTACAGAATGCCGAGATTACT-3; Reverse: 5-GGATGC TGGTGTCAACAGATG-3), and GATA-3 (Forward: 5-GCG GGCTCTATCACAAAATGA-3; Reverse: 5-GCTCTCCTGGCTGCAGACAGC-3 (Integrated DNA Technologies (IDT) Coralville, IA, USA) [54]. Expression levels were normalized by Δct using ubiquitin-conjugating enzyme (UBE2D2) as housekeeping gene [55] (Forward: 5-AGCTACAATAATGGGGCCAA-3; Reverse: 5- TGAAGGGGTAATCTGTTGGG-3) (IDT) and expressed such as relative units (RU).

### Statistical Analysis

Statistical analyses were done using GraphPad Prism 5 (San Diego, CA, USA). Comparisons of medians among groups were performed by the Wilcoxon or Kruskal-Wallis test and Dunńs post-test. Correlation was made with spearman test, *p* values lower than 0.05 were considered statistically significant.
